# A unique case of acute bilateral internal iliac deep vein thrombosis leading to right iliofemoral venous outflow obstruction

**DOI:** 10.1016/j.jvscit.2022.10.011

**Published:** 2022-11-15

**Authors:** Christina M. Melian, Stefanos Giannopoulos, Mary Lee, Angela A. Kokkosis

**Affiliations:** a Division of Vascular and Endovascular Surgery, Department of Surgery, Stony Brook University Hospital, Stony Brook, NY; b Jerry Pettis Memorial Veterans Hospital, VA Loma Linda Healthcare System, Loma Linda, CA

**Keywords:** Deep venous thrombosis, Hypogastric vein, Iliofemoral, Internal iliac vein, Phlegmasia, Thrombectomy

## Abstract

Venous thromboembolism has been associated with high morbidity and mortality, with a cost burden for the U.S. health care system owing to secondary complications such as pulmonary embolism and post-thrombotic syndrome. The current standard of therapy for acute deep vein thrombosis (DVT) is anticoagulation. For patients with venous outflow obstruction of the iliac vein system, several minimally invasive recanalization techniques are now available. In the present report, we have described a case of bilateral internal iliac DVT that had progressed to right-sided iliofemoral DVT in a young athletic adult, in the absence of anatomic abnormalities, that was treated with thrombolysis-free mechanical thrombectomy.

Venous thromboembolism, including deep vein thrombosis (DVT) and pulmonary embolism (PE), affects 1 in 1000 adults annually, with an estimated annual mortality of 60,000 to 100,000 persons and healthcare cost as high as $5 to $8 billion.^1^, ^2^, ^3^ Proximal iliofemoral DVT accounts for 25% of all cases of lower extremity DVT and has been associated with an increased risk of PE, limb malperfusion, and post-thrombotic syndrome (PTS).^4^ Right-sided DVT, in particular, has been shown to result in higher rates of subsequent PE.

The mainstay treatment of acute DVT is anticoagulation. Although thrombolysis (either systemic or catheter directed), with or without revascularization, is an option for DVT treatment, it has typically been reserved for extensive thrombus and phlegmasia, given the increased risk of major bleeding events.^5^ More recent literature has supported the use of early minimally invasive mechanical thrombectomy for the prevention of PTS and long-term sequelae; however, randomized trials are still underway.^6^

In the absence of any anatomic abnormalities or hypercoagulable disorder, right-sided proximal DVT is rare, especially in the setting of bilateral internal iliac vein DVT. In the present report, we have described a case of bilateral internal iliac vein DVT that had progressed to symptomatic right-sided iliofemoral DVT in an otherwise healthy young athlete, who had presented with phlegmasia and was treated with mechanical thrombectomy. The patient provided written informed consent for the report of her case details and imaging studies. Our goal was to summarize the current literature, report our experience with this relatively new endovascular approach, and discuss the role of thrombolysis-free mechanical thrombectomy in the setting of extensive thrombus burden.

## Methods

### Patient characteristics

**Fig 1:**
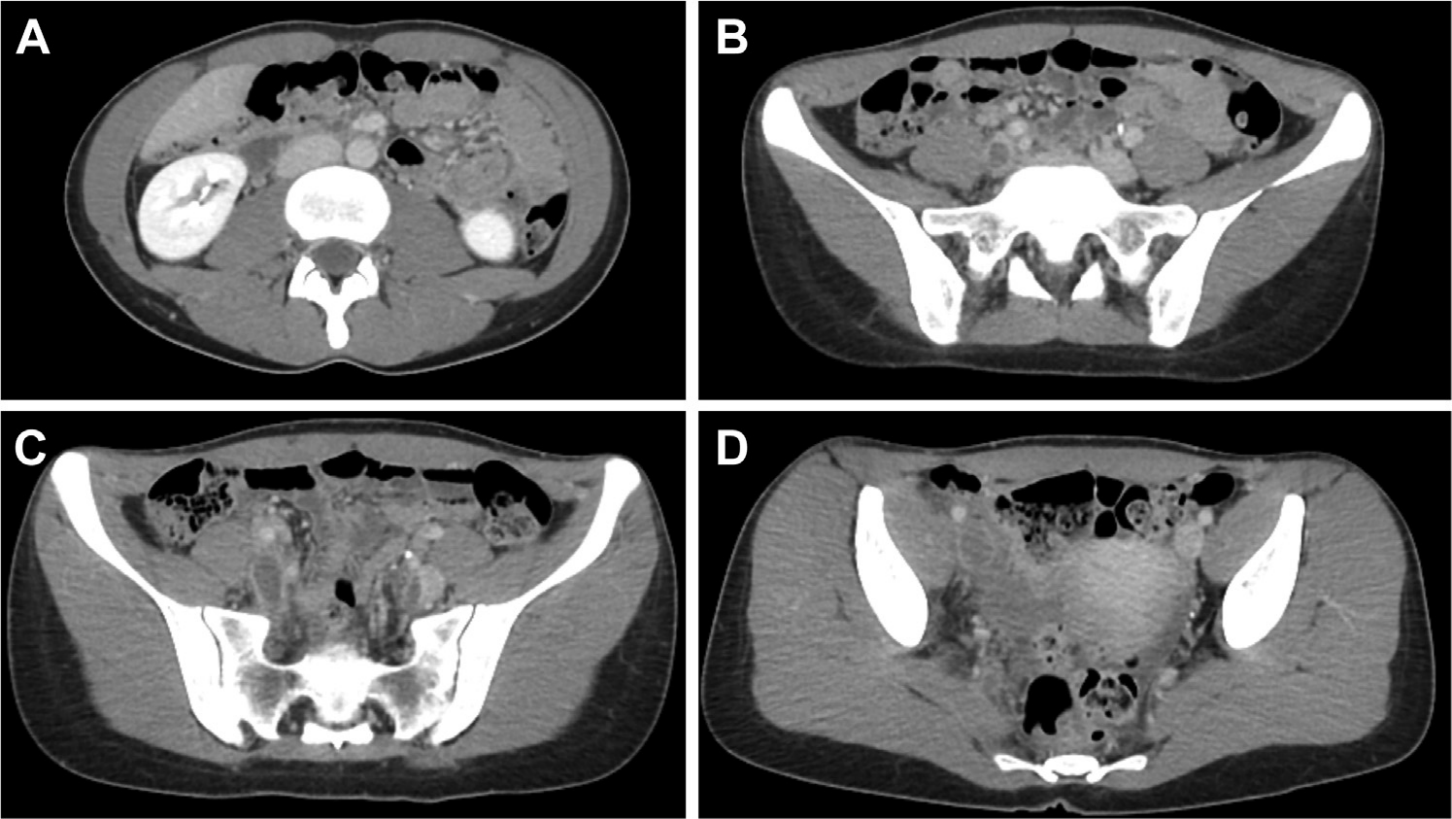
Computed tomography venogram demonstrating no thrombus in the inferior vena cava **(A)**, occlusive thrombus within bilateral internal iliac veins **(B** and **C)**, partial occlusion of the left common iliac vein due to propagation of the internal iliac thrombus, and complete occlusion of the right common iliac and external iliac veins **(C** and **D)**.

A 22-year-old, athletic woman had presented to the emergency department with acute-onset right lower extremity swelling and bluish skin discoloration 1 day after presenting to an outpatient orthopedic clinic with complaints of right hip pain. She was a long-distance collegiate runner and reported progressively worsening hip pain of several weeks’ duration despite deescalation of training, with dyspnea on exertion. She had a history of polycystic ovarian syndrome and was taking combined oral contraceptives at presentation. She denied any family history of coagulopathy or recent COVID-19 (coronavirus disease 2019) illness or exposure. Imaging with venous ultrasound and computed tomography venography demonstrated occlusive thrombus within the bilateral internal iliac veins, partial occlusion of the left common iliac vein, complete occlusion of the right common iliac and external iliac veins (Fig 1) due to propagation of the internal iliac vein thrombus, and subsegmental bilateral PEs. The oral contraceptives were discontinued, and patient started therapeutic anticoagulation with continuous heparin infusion.

### Endovascular technique

**Fig 2:**
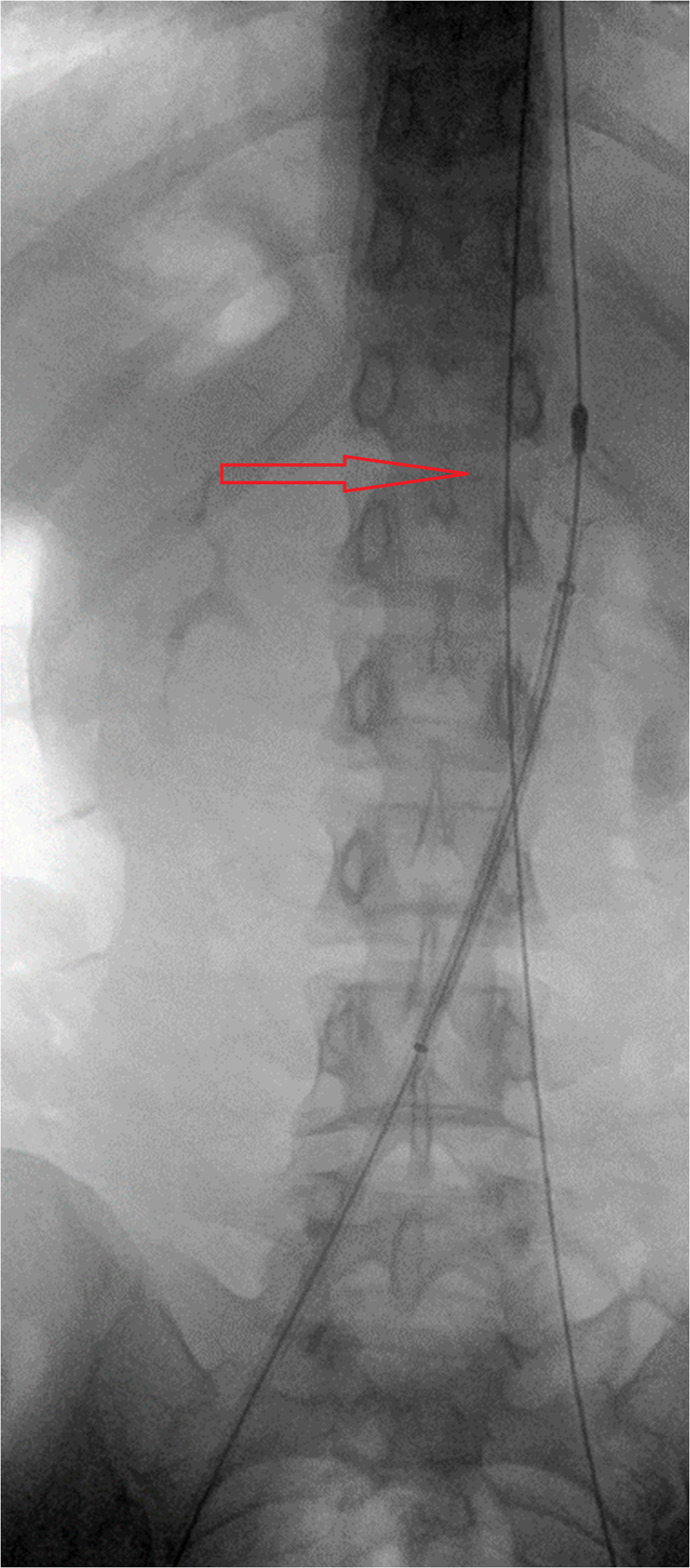
An Inari disc (Inari Medical) was used as a temporary filter protection device (*red arrow*).

**Fig 3:**
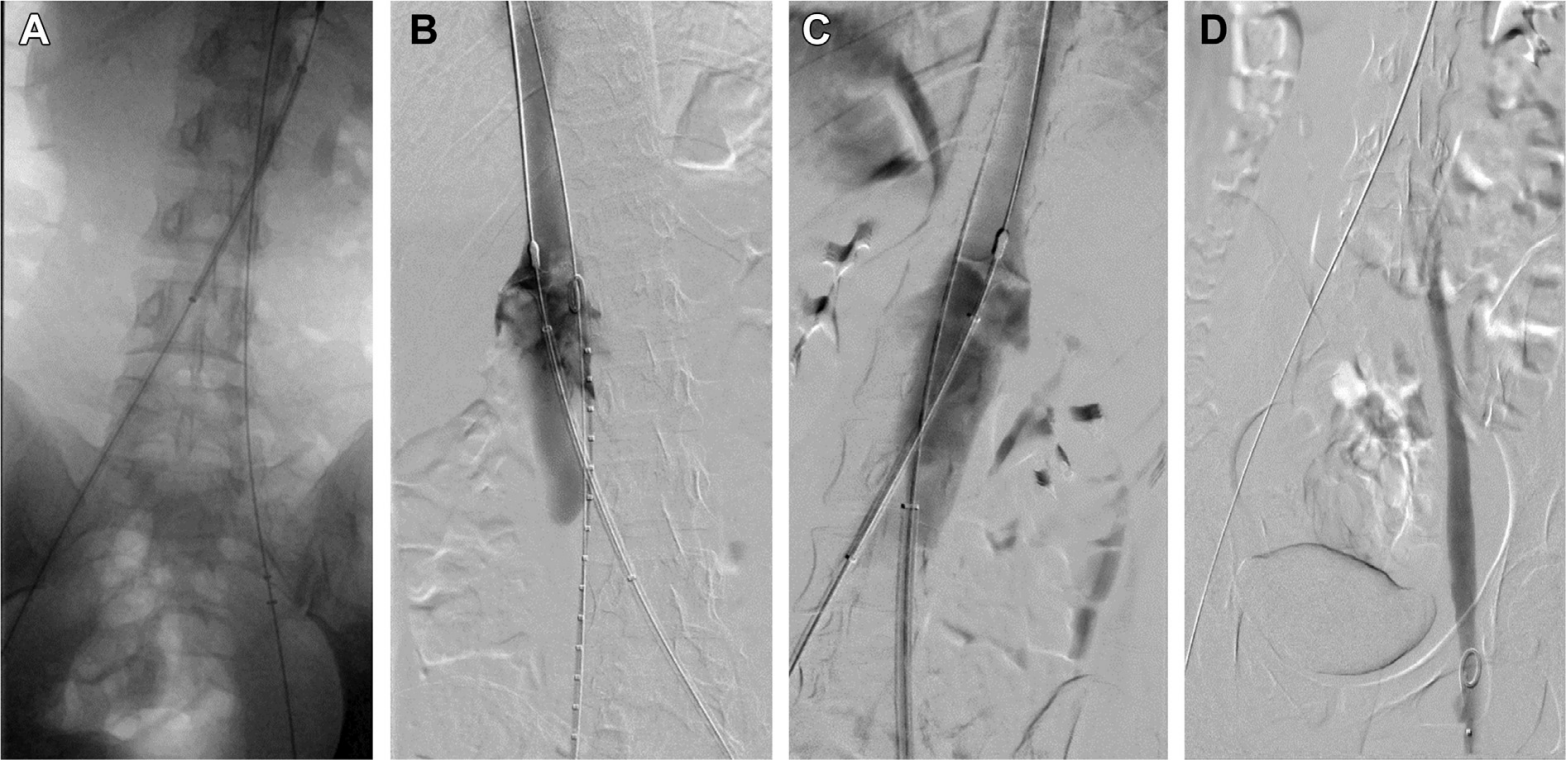
**A,** Inari ClotTriever mechanical thrombectomy device (Inari Medical) was advanced through the right popliteal access, and multiple passes were made until no thrombus was retrieved. **B,** Residual filling defects at level of protection disc suggestive of thrombus after mechanical thrombectomy. **C,** Suction thrombectomy of the inferior vena cava below the disc using the Penumbra CAT 12 lightning aspiration system (Penumbra, Inc). **D,** Completion venogram showing no residual thrombus.

Bilateral popliteal veins were accessed with ultrasound guidance, and 11F and 16F sheaths were placed in the left and right popliteal veins, respectively. An XL (19-25 mm) Inari disc (Inari Medical, Irvine, CA) was placed over the wire just below the renal confluence via the contralateral popliteal fossa as a temporary, extra filter protection device, with access maintained with a guidewire via the ipsilateral side (Fig 2). Intravascular ultrasound (IVUS) was used to identify the exact extent of the thrombus from the right popliteal vein access. The right common femoral, external iliac, and common iliac veins were thrombosed, without involvement of the inferior vena cava. The Inari ClotTriever mechanical thrombectomy device (Inari Medical) was advanced through the right popliteal access, and multiple passes were made until no thrombus was retrieved (Fig 3, *A*). Repeated venography demonstrated residual filling defects at the level of the protection disc suggestive of thrombus (Fig 3, *B*). Thus, a CAT 12 lightning aspiration system (Penumbra, Inc, Alameda, CA) was advanced through the right popliteal access, and successful suction thrombectomy of the inferior vena cava below the disc was performed (Fig 3, *C*).

Once all the thrombus had been removed, repeat venography and IVUS were performed to confirm the absence of residual thrombus before removing the protective disc. The disc was retrieved, and IVUS and completion venography (Fig 3, *D*) were performed, with no residual thrombus found. The sheaths were removed from both groins, and hemostasis was achieved with manual pressure. The right leg was wrapped with compression bandages up to the thigh, and patient was given full-dose anticoagulation therapy (enoxaparin 55 mg twice daily for 30 days, followed by rivaroxaban 20 mg daily thereafter). The patient was discharged home the next day and advised against the use of oral contraceptives.

## Results

Overall, the procedure was successful, with IVUS and angiographic imaging showing complete blood flow restoration in the right iliofemoral venous outflow system. The total intraoperative fluoroscopy time was 35.7 minutes, with a radiation dose of 55.6 mGy. The estimated blood loss was 100 mL. No perioperative complications were observed. Within 3 to 5 days after the index procedure, the edema had completely resolved, and the patient reported complete symptomatic relief. At the 2-week follow-up visit, the patient remained free of symptoms and was able to return to her normal daily activities. In the interim, she had undergone a hematology evaluation, and the hypercoagulable findings were negative.

## Literature review

The PubMed/Medline, Scopus, and Cochrane Central databases and presentations at conferences were searched for English-language prospective or retrospective analyses reporting on iliofemoral DVT endovascular revascularization techniques. The search was conducted up to June 2022. The keywords included in the search algorithm were as follows: iliofemoral, deep vein thrombosis, thrombectomy. Data were extracted for the following variables: population demographics, treatment modality, lesion location, follow-up duration, primary outcomes, and major conclusions from each study. Because of the between-study differences in design, comparisons made, and reporting methods, the findings were summarized from each study but the results were not pooled. Data from contemporary studies are summarized in the Table.^7^, ^8^, ^9^, ^10^, ^11^, ^12^

**Table:** Studies investigating outcomes of mechanical thrombectomy for acute-onset iliofemoral deep vein thrombosis (DVT)

Investigator	Patients, No.	Study design	Population	Age, years	Demographics	Revascularization	Thrombolysis	Follow-up, months	Main results	Conclusion
Dexter et al,^7^ 2022	250	Multicenter, prospective, single-arm study (CLOUT registry)	Acute and nonacute lower extremity DVT	Median, 62	Contraindications to thrombolytic agents, 40%	Mechanical thrombectomy with anticoagulation at discretion of operator; balloon angioplasty with/without stenting performed at discretion of operator	No	6	86% had ≥75% thrombus removal; 99.6% of patients were treated in single session; 24% of patients had PTS during follow-up	Mechanical thrombectomy showed promising results with significant improvement in symptoms and PTS
Jolly et al,^8^ 2022	96	Retrospective review	Unilateral iliofemoral DVT	Mean, 55	Female, 70%; underlying malignancy, 8.3%; recent surgery, 24%; prior DVT, 16.7%; contraindications to thrombolytic agents, 40%	Mechanical thrombectomy and therapeutic anticoagulation for all patients; additional common iliac vein stenting for 39%, with clopidogrel 75 mg for 60 days after procedure	No	1	97% had ≥75% thrombus removal; 2% had symptomatic PE; no mortality or major bleeding events	Mechanical thrombectomy was safe and effective for patients with acute iliofemoral DVT in this real-world study
Raskin et al,^9^ 2021	1	Case series	Unilateral iliofemoral DVT	50	Female patient with heart failure, lower extremity swelling, and pain	Mechanical thrombectomy with 24-hour thrombolysis and therapeutic anticoagulation	Yes	3	Complete resolution of symptoms at follow-up	Mechanical thrombectomy was safe and effective for patients with acute iliofemoral DVTs, with a minimal bleeding risk and a short hospital stay
Raskin et al,^9^ 2021	1	Case series	Unilateral iliofemoral DVT	64	Male patient with history of chronic DVT and phlegmasia cerulea doles	Mechanical thrombectomy, balloon angioplasty, and therapeutic anticoagulation	No	2	Complete resolution of symptoms at follow-up	
Raskin et al,^9^ 2021	1	Case series	Unilateral iliofemoral DVT	64	Male patient with history of recurrent DVT, proximal lower extremity swelling and pain	Mechanical thrombectomy, stenting	No	2	Complete resolution of symptoms at follow-up	
Raskin et al,^9^ 2021	1	Case series	Unilateral iliofemoral DVT	51	Female patient taking oral contraceptives, history of hypopituitarism and lupus; anticoagulant therapy; lower extremity pain and swelling	Mechanical thrombectomy and therapeutic anticoagulation	No	1	Complete resolution of symptoms at follow-up	
Shah et al,^10^ 2021	15	Retrospective review	Unilateral iliofemoral DVT	Mean, 59	Female patients, 5; symptomatic at presentation, 14; prior DVT, 13; preexisting IVC filter, 8	Mechanical thrombectomy with ClotTriever and FlowTriever (n = 8) or ClotTriever (n = 5) or FlowTriever (n = 2) alone	No	1-6	Technical success in 13 patients; 1 had nonocclusive thrombus densely adherent to preexisting IVC filter; 1 had chronic rubbery clot in IVC that could not be cleared	ClotTriever and FlowTriever showed promising results in treatment of IVC thrombosis without thrombolytic agents and with no major bleeding events or requirement for intensive care unit stay
Benarroch-Gampel et al,^11^ 2020	12	Retrospective review	Unilateral iliofemoral × 10, iliocaval ×1, femoropopliteal ×1	Mean, 55.6	Female, 6; underlying malignancy, 4; recent surgery, 3; phlegmasia cerulea dolens, 1; disabling pain and swelling, 11	Mechanical thrombectomy	No	Mean, 4 (1-10)	11 reported symptom resolution; 2 developed recurrent occlusive DVT	Mechanical thrombectomy was safe and effective for removing large volumes of lower extremity acute thrombus in a single session without lytic therapy, intensive care unit admission, or repeat intervention required
Pezold et al,^12^ 2020	1	Case report	Unilateral iliofemoral DVT and PE	10	Female patient with granulomatosis with polyangiitis, leg edema, and venous claudication	Mechanical thrombectomy and therapeutic anticoagulation	No	1	No residual thrombus; resolution of symptoms	Mechanical thrombectomy of iliofemoral DVT in a pediatric patient with ANCA-associated vasculitis via percutaneous, nonpharmacologic treatment is feasible, with promising results

*ANCA,* Antineutrophil cytoplasmic antibodies; *CLOUT,* ClotTriever outcomes; *IVC,* inferior vena cava; *PE,* pulmonary embolism; *PTS,* post-thrombotic syndrome.

## Discussion

We have described the case of a young woman with acute on chronic bilateral internal iliac DVT that had led to right iliofemoral venous outflow obstruction causing phlegmasia of the right leg that was eventually treated successfully with endovascular thrombectomy with a ClotTriever (Inari Medical). The patient reported the immediate relief of symptoms, supporting the use of thrombolysis-free mechanical thrombectomy for unusual cases of iliofemoral DVT.

The pathophysiology of DVT is best explained by the Virchow triad of hypercoagulability, venous stasis, and endothelial damage.^1^^,^^13^ Predisposing clinical conditions, such as surgery, trauma, malignancy, prolonged immobility, pregnancy, obesity, advancing age, and a history of DVT, have all been shown to increase the risk of DVT.^1^ Although physical immobility has been described as a risk factor for DVT, some studies have suggested that competitive athletes could also have a high risk of DVT.^14^, ^15^, ^16^ More recent studies have suggested that COVID-19 exposure might play a role in DVT formation in critically ill patients.^17^

The mainstay treatment of lower extremity DVT is anticoagulation, with thrombectomy reserved for extensive clots. The American Society of Hematology published guidelines for the optimal use of anticoagulant drugs for the prevention and treatment of DVT.^18^ However, the use of anticoagulation therapy has not demonstrated a significant benefit in reducing the risk of subsequent PTS after DVT. Recent studies have shown early mechanical thrombectomy to reduce the risk of PTS and post-DVT sequela.^18^^,^^19^ Quinn et al^19^ presented a case of left-sided lower extremity DVT treated with the ClotTriever (Inari Medical), supporting its use in alleviating extensive clot burden. However, given the rarity of right-sided iliofemoral venous outflow, limited literature is available regarding the use of mechanical thrombectomy in the management of right-sided thromboses.

The CaVenT (post-thrombotic syndrome after catheter-directed thrombolysis for deep vein thrombosis) clinical trial (ClinicalTrials.gov identifier, NCT00251771) included 176 individuals with high proximal leg DVT from 20 hospitals in Norway.^20^ These patients had been treated conservatively with compression stockings and anticoagulation therapy or had undergone catheter-directed thrombolysis.^20^ The study showed that the incidence of PTS was significantly lower in the thrombolysis group than in the control group, although the quality of life scores (Villalta score pf ≥15) were similar between the two groups during follow-up.^20^ Thus, the investigators underlined the need for investigation regarding whether endovascular thrombolysis will provide clinical benefit that outweighs the higher periprocedural risk of bleeding.

A similar, multicenter, randomized clinical trial, with larger population size (692 patients) by Vedantham et al^21^ (ATTRACT [acute venous thrombosis: thrombus removal with adjunctive catheter-directed thrombolysis]; ClinicalTrials.gov identifier, NCT00790335) showed that the addition of pharmacomechanical catheter-directed thrombolysis to anticoagulation resulted in a higher risk of perioperative major bleeding (1.7% vs 0.3%), without lowering the risk of PTS during 24 months of follow-up (47% vs 48%). Therefore, some could argue against the routine use of catheter-directed thrombolysis because of the associated high risk of bleeding and questionable effects on long-term quality of life. In the present report, we have demonstrated that thrombolysis-free mechanical thrombectomy can safely be used for the treatment of this clinical entity, with promising acute luminal gain and blood flow restoration. IVUS can provide valuable information intraoperatively and facilitate clinical decision-making.^22^ Nonetheless, additional research is needed to better optimize the current treatment algorithms for extensive proximal iliofemoral DVT.

## Conclusions

Right-sided iliofemoral DVT in the absence of anatomic abnormalities and/or hypercoagulable disorders is rare but has been associated with secondary complications that correlate with high morbidity and mortality and usage of health care resources. The current literature does not support the addition of thrombolysis to anticoagulation for a lower risk of PTS. In addition, it has been associated with higher risk of major bleeding. Our findings have demonstrated that thrombolysis-free mechanical thrombectomy is appropriate for extensive proximal iliofemoral venous outflow obstruction, limiting the risk of major bleeding and showing promising favorable outcomes. Nonetheless, further research is warranted to help determine appropriate criteria for the application of contemporary revascularization techniques for proximal DVT.
